# Pien-Tze-Huang alleviates CCl_4_-induced liver fibrosis through the inhibition of HSC autophagy and the TGF-β1/Smad2 pathway

**DOI:** 10.3389/fphar.2022.937484

**Published:** 2022-09-16

**Authors:** Yuqin Zhang, Liping Hua, Chunfeng Lin, Mingzhou Yuan, Wei Xu, Anand Raj D., Baskar Venkidasamy, Carlos L. Cespedes-Acuna, Shivraj Hariram Nile, Guohong Yan, Haiyin Zheng

**Affiliations:** ^1^ Pharmacy College, Fujian University of Traditional Chinese Medicine, Fuzhou, Fujian, China; ^2^ College of Integrative Medicine, Fujian University of Traditional Chinese Medicine, Fuzhou, Fujian, China; ^3^ Department of Biotechnology, Karpagam Academy of Higher Education (Deemed to be University), Coimbatore, Tamil Nadu, India; ^4^ Department of Oral and Maxillofacial Surgery, Saveetha Dental College and Hospitals, Saveetha Institute of Medical and Technical Sciences (SIMATS), Saveetha University, Chennai, Tamil Nadu, India; ^5^ Plant Biochemistry and Phytochemical Ecology Lab, Basic Sciences Department University of Bio Bio, Chillan, Chile; ^6^ School of Pharmaceutical Science, Zhejiang Chinese Medical University, Hangzhou, Zhejiang, China; ^7^ Affiliated People’s Hospital of Fujian University of Traditional Chinese Medicine, Fuzhou, Fujian, China

**Keywords:** Pien-Tze-Huang, liver fibrosis, hepatic stellate cell, autophagy, transforming growth factor-β1, matrix metalloproteinase

## Abstract

**Ethnopharmacological relevance:** Pien-Tze-Huang (PZH)—a traditional Chinese medicine (TCM) compound—has been employed to treat various liver inflammation and tumors for over 10 decades. Interestingly, most of the pharmacological effects had been validated and explored toward liver ailment along with pro-inflammatory conditions and cancer at the cellular and molecular level to date.

**Aim of the study:** The present study aimed to investigate the therapeutic effect of PZH on autophagy and TGF-β1 signaling pathways in rats with liver fibrosis and hepatic stellate cell line (HSC).

**Materials and methods:** Male SD rats with carbon tetrachloride (CCl4)-induced liver fibrosis were used as the animal model. Next, PZH treatment was given for 8 weeks. Afterward, the therapeutic effects of PZH were analyzed through a hepatic tissue structure by hematoxylin-eosin (H&E), Van Gieson (VG) staining, and transmission electron microscopy (TEM), activity of ALT and AST by enzyme-associated immunosorbent assay as well. Subsequently, mRNA and protein expression were examined by quantitative polymerase chain reaction (qPCR), Western blotting, and immunohistochemistry (IHC). Then, the cell vitality of PZH-treated HSC and the expression of key molecules prevailing to autophagy were studied *in vitro.* Meanwhile, SM16 (a novel small molecular inhibitor which inhibits TGFβ-induced Smad2 phosphorylation) was employed to confirm PZH’s effects on the proliferation and autophagy of HSC.

**Results:** PZH pharmacologically exerted anti-hepatic fibrosis effects as demonstrated by protecting hepatocytes and improving hepatic function. The results revealed the reduced production of extracellular collagen by adjusting the balance of matrix metalloproteinase (MMP) 2, MMP9, and tissue inhibitor of matrix metalloproteinase 1 (TIMP1) in PZH-treated CCl4-induced liver fibrosis. Interestingly, PZH inhibited the activation of HSC by down-regulating TGF-β1 and phosphorylating Smad2. Furthermore, PZH down-regulated yeast Atg6 (Beclin-1) and microtubule-associated protein light chain 3 (LC3) toward suppressing HSC autophagy, and PZH exhibited similar effects to that of SM16.

**Conclusion:** To conclude, PZH alleviated CCl4-induced liver fibrosis to reduce the production of extracellular collagen and inhibiting the activation of HSC. In addition, their pharmacological mechanisms related to autophagy and TGF-β1/Smad2 signaling pathways were revealed for the first time.

## Introduction

Liver fibrosis is a characteristic of chronic liver diseases in response to a pathological change of wound-healing caused by chronic liver impairment, mainly encompassing virus invasion, alcoholism, and metabolic abnormalities (Perumpail et al., 2017; Asrani et al., 2019; Wang et al., 2019). Liver diseases are ultimately characterized by an excessive amassment of extracellular matrix (ECM) components and eventually develop into lethal complications such as hepatic sclerosis and malignancy that account for 3.5% of deaths worldwide (Moon et al., 2020). However, the reversibility of liver fibrosis has been well characterized; still, there exists a paucity of therapeutic options toward liver fibrosis irrespective of etiologies. It was reported that hepatic stellate cells are down-regulated in normal liver tissue (Xia et al., 2020). Activating the hepatic stellate cell (HSC) had been claimed as a key event in liver fibrogenesis (Yin et al., 2020). Once triggered by injury signals, the HSC transform from a normal “stationary state” to the “active state,” which is possibly manifested through loss of lipid droplets, appearance of myofibroblast-like phenotype, plus increased production of alpha-smooth muscle actin (α-SMA), type I collagen, and matrix metalloproteinases (MMPs) along with tissue inhibitor of matrix metalloproteinases (TIMPs), ultimately contributing to the progressive accumulation of fibrillar ECM in liver tissues sequentially (Higashi et al., 2017; Huang et al., 2017). On the other hand, recent reports suggest the removal of activated HSC *via* apoptosis or phenotypic regression to a static state to slow down or reverse liver fibrosis development (Elsharkawy et al., 2005; Campana and Iredale, 2017).

Autophagy, which plays an important role in liver diseases by means of defined death and survival supporting functions whereby organelles, proteins, and lipid droplets are degraded and recycled under physiological and pathological conditions (Sekar and Thirumurugan, 2022). Based on the modes of substrate transport to lysosomes, autophagy was divided into categories such as macroautophagy, microautophagy, and chaperone-mediated autophagy (Czaja et al., 2013). It is well-known that autophagy begins with the formation of an autophagosome (bilayer membrane) which contains cellular components and then fuses with lysosomes, thereby degrading the autophagic substrate (Liu et al., 2022). The origin of the autophagosome usually depends on the host with autophagy-related genes (ATGs) (Yu et al., 2022). Rising pieces of evidence uncovering the alteration in autophagy are strongly related to the pathogenesis of a wide range of diseases, including various liver diseases (Czaja et al., 2013; Pietrocola et al., 2013). Also, the disappearance of retinoid lipid droplets stands as the definite feature of HSC activation under the circumstance of liver fibrosis. Moreover, studies have reported that the energy provided through autophagy for activating HSC is produced by digesting lipid droplets often termed “lipophagy” (Thoen et al., 2011; Hernández-Gea and Friedman, 2012). Based on these findings, autophagy is considered as a potent pathway which facilitates HSC activation and liver fibrosis.

Transforming growth factor-β1 (TGF-β1) is a well-known pro-fibrogenic factor predominantly produced by macrophages and then activating the HSC in liver chronic inflammation. TGF-β1/Smad signaling is one of the key pathways participating in the activation of HSC and formation of liver fibrosis (Fabregat and Caballero-Diaz, 2018). The mechanism starts with binding to type I TGF receptor (TGFR) along with the recruitment of TGFRII, and also the phosphorylation of its downstream substrate, Smad2/3 proteins. Next, they combine with Smad4 to form a complex which transposes into the nucleus and bonds various transcription factors to regulate gene transcription, such as type I collagen, α-SMA, MMPs, and TIMPs in the HSC (Xu et al., 2016; Dewidar et al., 2019). Off note, TGF-β1/Smad pathway also modulates autophagy-related genes such as Beclin-1 (Pan et al., 2015). Many studies had revealed that multiple natural medicines will possibly combat liver fibrosis by adapting to the TGF-β1 signaling pathway (Peng et al., 2013; Liu et al., 2019; Ma et al., 2019).

Pien-Tze-Huang (PZH), a recognized TCM recommended 450 years ago during the Ming Dynasty, has been utilized as a folk cure for several forms of cancer in China and Southeast Asia for centuries (Shen et al., 2012) as posture prestigious Chinese patent medicine with antineoplastic, anti-inflammation, anti-oxidation, hepatocyte protection, and other functions. The formula of PZH is made of four TCM ingredients, *i.e*., Panax notoginseng (Araliaceae; Notoginseng Radix et Rhizoma; Sanqi in Chinese; 85%), Moschus (Cervidae; excretion of *Moschus berezovskii* Flerov; Shexiang in Chinese; 3%), Bovis Calculus (Bovidae; *Bos Taurus domesticus* Gmelin’s dry gallstones; Niuhuang in Chinese; 5%), and snake gall (Colubridae; snake gall bladder; Shedan in Chinese; 7%). It is officially used to treat acute and chronic hepatic diseases (Lee et al., 2002; Chan et al., 2004; Xu and Yu, 1994; Chinese Pharmacopoeia Commission, 2020).

Our previous study demonstrated the mitigative effect of PZH on experimental liver fibrosis on bounding up with the regulation of HSC activation and many inflammatory signaling [such as tumor necrosis factor (TNF)-α, nuclear factor kappa-light-chain-enhancer of activated B (NF-κB)] (Zheng et al., 2019). Nevertheless, its anti-fibrotic molecular mechanism was not fully clarified. Therefore, this study aimed to explore a well-established rat liver fibrosis model induced by carbon tetrachloride (CCl_4_) and cultured HSC. Furthermore, it aimed to reveal the role of PZH on crucial signaling pathways involved in fibrogenesis, such as autophagy and TGF-β1/Smad signaling.

## Materials and methods

### Drug and reagents

Colchicine was procured from Xishuangbanna Pharmaceutical Co., Ltd. (Jinghong, China, Chinese FDA approval No. 170306). CCl_4_ was obtained from Sinopharm Chemical Reagent Co., Ltd. (Shanghai, China) and dimethylsulfoxide (DMSO) was acquired from Sigma-Aldrich (St. Louis, MO, United States). SM16 was purchased from MedChemExpress (Shanghai, China). Primary anti-collagen I, α-SMA MMP-2, MMP-9, TIMP-1, LC3 I/II, Beclin-1, TGF-β1, Smad2, and p-Smad2 were provided by Abcam (Cambridge, Cambs, United Kingdom, Nos. Ab92536, Ab58803, Ab61224, Ab92486, and Ab33875), Merck (Kennedy, New Jersey, United States, No. ABT123), and Cell Signaling Technology, Inc. (Danvers, MA, United States, Nos. 19245, 12741, 3495, and 18338). In addition, the commercial kits and other reagents were purchased from Nanjing Jiancheng Biotechnology Research Institute (Nanjing, China).

### Preparations of Pien-Tze-Huang

PZH is a precious Chinese patent medicine acquired and authenticated by the sole manufacturer Zhangzhou PZH Pharmaceutical Co., Ltd. (Zhangzhou, China, Chinese FDA approval No. Z35020242). Ultra-high-performance liquid chromatography (UHPLC) hyphenated with mass spectrometry has been employed in the chemical analysis of traditional Chinese medicine (Zhu et al., 2013). In this present study, 10 target compounds (notoginsenoside R1, ginsenoside Rb1, ginsenoside Rg1, ginsenoside Rg3, cholic acid, deoxycholic acid, hyodeoxycholic acid, ursodeoxycholic acid, chenodeoxycholic acid, sodium taurochenodeoxycholate, sodium tauroursodeoxycholate, and muscone) were measured as the quantity of PZH by UHPLC–QqQ-MS. The HPLC chromatogram is shown in Supplementary Figure S1.

Next, PZH extract for treating cells was prepared before use by means of dissolving PZH powder in dimethylsulfoxide (DMSO) with a 50 mg/ml concentration. Next, the working solution was prepared by diluting the stock solution in the culture medium and then filtered (Li et al., 2022a). PZH suspension for treating rats was prepared by dissolving the powder in normal saline and was used directly after an ultrasound.

### Animals and treatment

A total of 40 6-week-old SD (Shanghai Slac Laboratory Animals Co., Ltd., Shanghai) male rats were kept in the SPF animal laboratory of the Animal Experimental Center of Fujian University of Traditional Chinese Medicine (the ethics approval number for the use of animals was 2019079). Subsequently, the feeding conditions of mice were as follows: temperature of 21°C–23°C, humidity of 55–75%, and a day and night cycle for 12 h. After 5 days of adaptive feeding, four groups of rats were prepared: 1) normal control group; 2) CCl_4_ group; 3) CCl_4_+PZH group; and 4) CCl4+colchicine group. Following the previously reported protocol, the rats were given 50% CCl_4_–peanut-oil solution twice a week for 8 weeks. Accordingly, the control with a normal group of rats was maintained. The rats in groups 3) and 4) were administered 0.15 g/kg PZH orally (As shown in Supplementary Figure S2, hematoxylin-eosin (H&E) and Van Gieson (VG) staining assay showed that PZH with doses 0.075, 0.15, and 0.3 g/kg could protect against CCl_4_–induced liver fibrosis, especially 0.15 and 0.3 g/kg. In light of these results, a concentration of 0.15 g/kg was used to further explore the effects and possible mechanisms of PZH against liver fibrosis *in vivo*) and 0.1 mg/kg of colchicine from the beginning of the trial as reported in previous studies. Meanwhile, the other two groups were given an equal volume of saline once a day for 8 weeks. Next, blood and livers were extracted 1 h after the final treatment in the 8th week for follow-up tests. All animals were used in accordance with the guidelines for the Care and Use of the Laboratory Animal by the National Institutes of Health.

### Enzyme-associated immunosorbent assay

Serum was obtained by centrifuging at 3,500 rpm and then detecting the activity of alanine aminotransferase (ALT) and aspartate aminotransferase (AST) at 510 nm (Infinite M200 Pro; Tecan, Männedorf, Switzerland). All the procedures performed in this study were in accordance with the manufacturer’s protocol. Eventually, the absorbance was recorded at 450 nm by using a microplate reader (Hadizadeh and Nasirian, 2022).

### Histological assessment

The fixed liver tissue was embedded, cut into 5 μm thick sections, and finally stained with HE and VG. Next, the three pathological sections were observed with an optical microscope and photographed with a video camera. The VG staining grade score was evaluated according to the grading criteria for hepatic fibrosis: no fibrosis, no necrosis of hepatocytes, 0 points; fibrous tissue hyperplasia, fibrosis limited in the portal area of porta hepatis, 1 point; hepatic fibrosis occurs around the portal area of porta hepatis, the fiber spacing formed, and liver lobule structure retained, 2 points; fibrous septa with liver lobular structure disorder, but no obvious cirrhosis, 3 points; false hepatic lobule formation, early cirrhosis, 4 points. It was calculated by using an automated image analyzer (Qwin, Leica) with a color scan.

### Transmission electron microscopy

To evaluate the ultrastructural changes and accurately evaluate autophagy, representative liver tissues were prepared for transmission electron microscopy (TEM) as previously described (Hernández-Aquino et al., 2019).

### Immunohistochemistry analysis

In brief, the 5 μm thick paraffin-embedded tissue sections were dewaxed and rehydrated. Next, antigen retrieval with citrate buffer and blocking was carried out with nonspecial binding sites with goat serum and then, the sections were incubated with a primary antibody and horseradish peroxidase-linked secondary antibody. Finally, the samples were incubated with diaminobenzidine (DAB) for the chromogenic reaction and then counterstained with hematoxylin. The results of the experiment were based on the three positive areas observed and the number of positive cells which turned brown was calculated by ImageJ IHC Profiler software and an automated image analyzer (Qwin, Leica) with a color scan in three independent experiments.

### Cell culture and treatment

The hepatic stellate cell line (HSC-T6) was procured from Sangon Biotech co., Ltd. (Shanghai China) and cultured in a complete medium. HSC-T6 cells within 2–5 generations of subculture were inoculated in 96-well or 6-well culture plates. Next, cell confluence reached 50–60% before the treatment. Subsequently, the powdery PZH was dissolved in DMSO in advance to obtain a 50 mg/ml storage solution and the medium was continuously diluted to working concentrations of 100, 200, 300, 400, and 500 μg/ml. Overall, there were six groups of cells: vehicle control group, TGF-β1 group, and TGF-β1+PZH groups with different concentrations. Henceforth, the TGF-β1 group was given the recommended concentration (5 μM) of TGF-β1 and incubated for 48 h. The TGF-β1+PZH groups were simultaneously treated with TGF-β1 (5 μM) and PZH, and the control group was given the corresponding medium for 48 h. Then, all these cells were examined for cell viability and protein expression.

Smad2 phosphorylation is a key mechanism in the mediation of autophagy activation. Furthermore, to accurately evaluate the effects of PZH on the proliferation and autophagy of the HSC while Smad2 phosphorylation is blocked, SM16 (200 nM, a novel small molecular inhibitor which inhibits TGFβ-induced Smad2 phosphorylation) was employed. Finally, cell viability and autophagy-related protein expression were examined.

### Cell proliferation detection

MTT staining was performed to detect the cell proliferation of HSC-T6. The steps are as follows: cells in different experimental groups were treated with PZH and/or TGF-β1 administrating, then MTT solution was added to each well for 4 h, and then the generated formazan was dissolved with DMSO and the absorbance was recorded at 570 nm. Cell activity was obtained by calculating the ratio of absorbance between the treatment groups and the control group for six independent experiments.

### Western blotting

Liver (cell protein) extract was assayed with a BCA kit according to the manufacturer’s protocols toward obtaining an accurate concentration of proteins and then added to 1/4 volume of protein buffer and denatured by heating. Aliquots of the samples (40 μg) were analyzed using SDS-PAGE gel. Next, the gel was transferred and then blocked and incubated with different corresponding primary antibodies overnight. Subsequently, the target membranes were incubated with a secondary antibody. Proteins were detected using the ECL kit and the results were analyzed using Image Lab analysis software (Bio-Rad Laboratories, Inc., Hercules, California, United States) for three replicates.

### Quantitative real-time polymerase chain reaction

We used the Trizol method (Trizol reagent) (Invitrogen, Barcelona, Spain) to extract the total RNA from quick-frozen tissues or cells and aliquots of the total RNA (1 μg) which was reverse-transcribed with the PrimeScript^®^ RT reagent kit (Takara Bio, Inc., Otsu, Japan). Subsequently, quantitative real-time PCR was carried out at the 7900 Real-Time PCR system (Applied Biosystems, Inc., Foster City, CA, United States) according to the instructions. The primer sequences required for the experiment are shown in Table 1. Relative mRNA expression levels of target genes were calculated according to the threshold cycle (Ct) value based on 2^−△△Ct^ formula using GAPDH as an internal control for sample normalization for three independent experiments.

**TABLE 1:** Primer sequence for real-time PCR.

Primer name	Primer sequence
GAPDH	F’ GCTTCACCACCTTCTT GATGTC
	R’ TGAACGGGAAGCTCACTGG
COI-1	F’ GTGAGACAGGCGAACAAGG
	R’ GGACCAGCAGGACCACTAT
α-SMA	F’ AGACCTTCAATGTCCCTGCCA
	R’ TGTAGGTGGTTTCGTGGATGC
MMP-2	F’ GCAACCACAACCAACTACGA
	R’ TACCAGTGTCAGTATCAGCATCA
MMP-9	F’ CAAGGACGGTCGGTATTGGAAG
	R’ AAACGAGTAACGCTCTGGGGAT
TIMP-1	F’ GCTAAGATGCTCAAAGGATTCG
	R’ GATGGCTGAACAGGGAAACACT
TGF-β1	F’ ATGAACCGACCCTTCCTGCT
	R’ CCTGGTTGTGTTGGTTGTAGAG
Smad2	F’ GCCGCCTCTGGATGACTATA
	R’ AAGCCGTCTACAGTGAGTGA

### Statistical analysis

All results of the experiment were shown as means ± SD. SPSS software 21.0 was used to analyze the data and one way ANOVA followed by a post-hoc LSD, and the Gaimshowell test was used to analyze the differences. The difference was designated as statistically significant when the *p*-value was <0.05.

## Results

### Pien-Tze-Huang ameliorated carbon tetrachloride-induced liver fibrosis in rats

**FIGURE 1:**
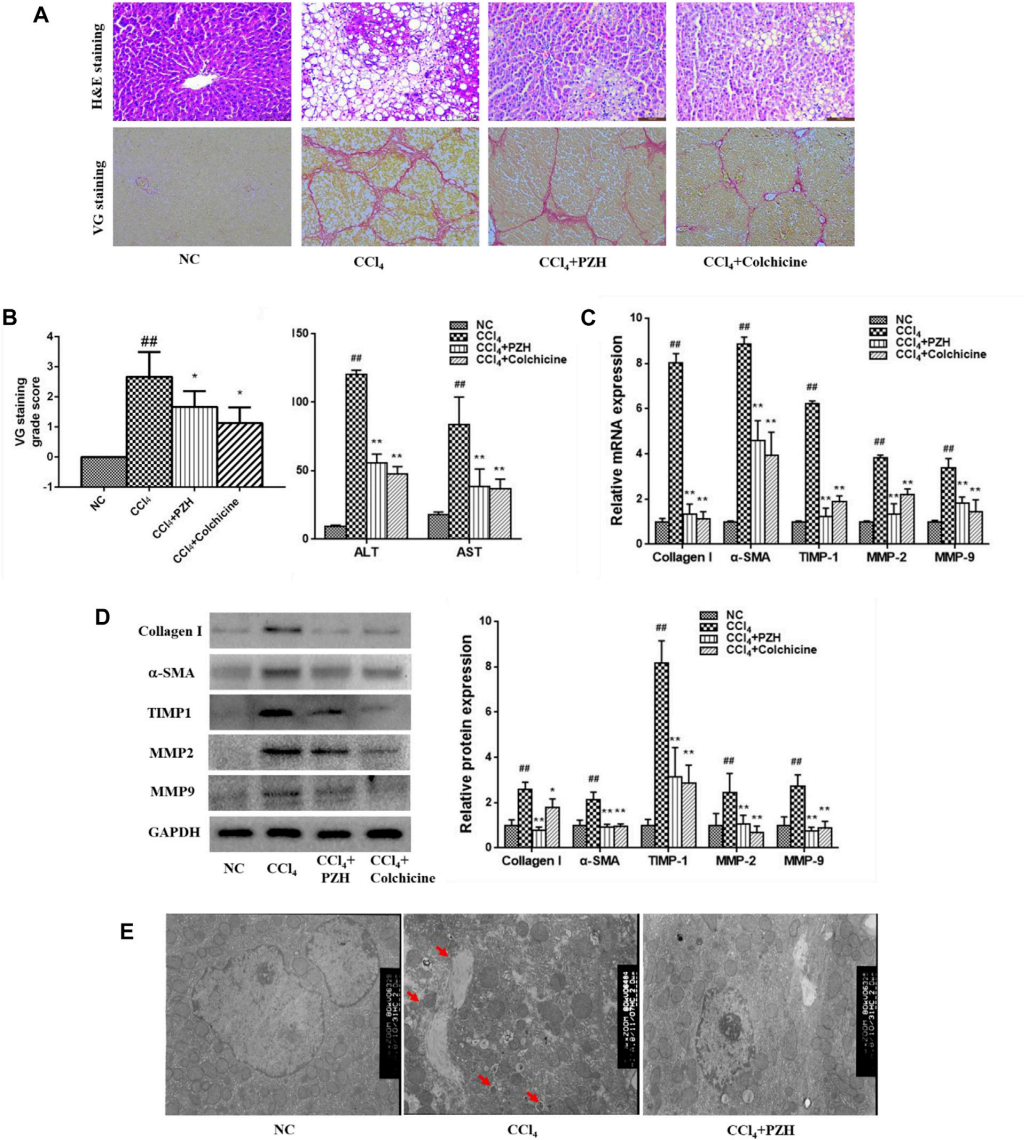
Effect of PZH on CCl_4_-induced liver fibrosis, HSC activation, and collagen deposition in rats. **(A)** Representative images of VG-stained liver sections (magnification×200); **(B)** VG staining grade score and serum transaminase levels by ELISA assay; **(C)** quantitative PCR detection of collagen I, α-SMA, MMP 2,9, and TIMP1 mRNA levels; **(D)** Western blot and densitometry analysis of collagen I, α-SMA, MMP 2,9, and TIMP1 protein levels. GAPDH was used as an internal control; **(E)** ultra-structural changes in the liver tissue of rats observed by TEM. Autophagosomes (red arrows) were observed. Images were taken at 5,000× magnification. ^##^ *p* < 0.01 versus NC group; ^**^ *p* < 0.01 versus CCl_4_ group.

The impact of PZH on the exposure of rat liver fibrosis toward CCl_4_ and H&E along with VG staining was executed. The obtained outcomes revealed the hepatic histological changes and collagen fibers in liver sections comprehensively. Also, rats in the model group displayed obvious vacuole degeneration and destruction of the normal hepatic lobule structure and the even reconstruction of the hepatic lobule structure. Interestingly, the level of collagen fiber production in liver tissues increased after 8 weeks of CCl_4_ administration. Figures 1A,B show the degree of difference in VG-stained liver sections with the control groups. Next, the cross-linking and segmentation of the fibers disrupted the lobular structure and formed a pseudo-lobule. In contrast, the point of hepatocyte injury in the PZH treatment group was significantly reduced. Next, the generation of collagen fibers in the liver was down-regulated with no obvious pseudo-lobule formation. Also, the function of PZH in relieving fibrosis in the liver was similar to that of colchicine, the positive control drug. Moreover, TEM images (Figure 1E) show a normal liver ultrastructure in the control group, where several mitochondria and typical nuclei can be observed, while livers derived from CCl_4_-treated rats showed disorganized mitochondria with loss of cristae, diminished endoplasmic reticulum, and presence of lipid droplets and autophagosomes (red arrows) as well. In the livers from rats treated with CCl_4_ plus PZH, almost all alterations were reversed, showing an appearance similar to that of the normal control group. On the other hand, ALT and AST are important serum indices of hepatic function. As shown in Figure 1B, the toxic effect of CCl_4_ resulted in the obvious elevation of ALT and AST in serum, which remarkably decreased after PZH treatment. These obtained results indicate that PZH had a favorable effect on protecting hepatocytes and improving hepatic function.

### Pien-Tze-Huang inhibited the activation of hepatic stellate cell and extracellular matrix deposition in liver fibrosis

Also, activated HSC leads to the production of large amounts of ECM, particularly type-I collagen. Therefore, the impact of PZH on the activation of the HSC and sediment of the ECM in addition to matrix-degrading enzymes was investigated *in vivo*. As shown in Figures 1C,D, the gene and protein expression levels of the α-SMA in activated HSC and collagen I in the fibrosis group were higher than those in the normal group; meanwhile, they were apparently down-regulated in both PZH and colchicine treatment groups. Additionally, MMP2 and 9 and TIMP1 were detected in liver tissues that were dominant proteases regulating the balance of ECM production and degradation. Furthermore, Western blotting and quantitative PCR results revealed the expression level of genes and proteins such as MMP2 and 9 and TIMP1. Meanwhile, their expression levels showed a significant decline after treatment with PZH and colchicine.

### Pien-Tze-Huang suppressed carbon tetrachloride-induced autophagy in liver fibrosis

**FIGURE 2:**
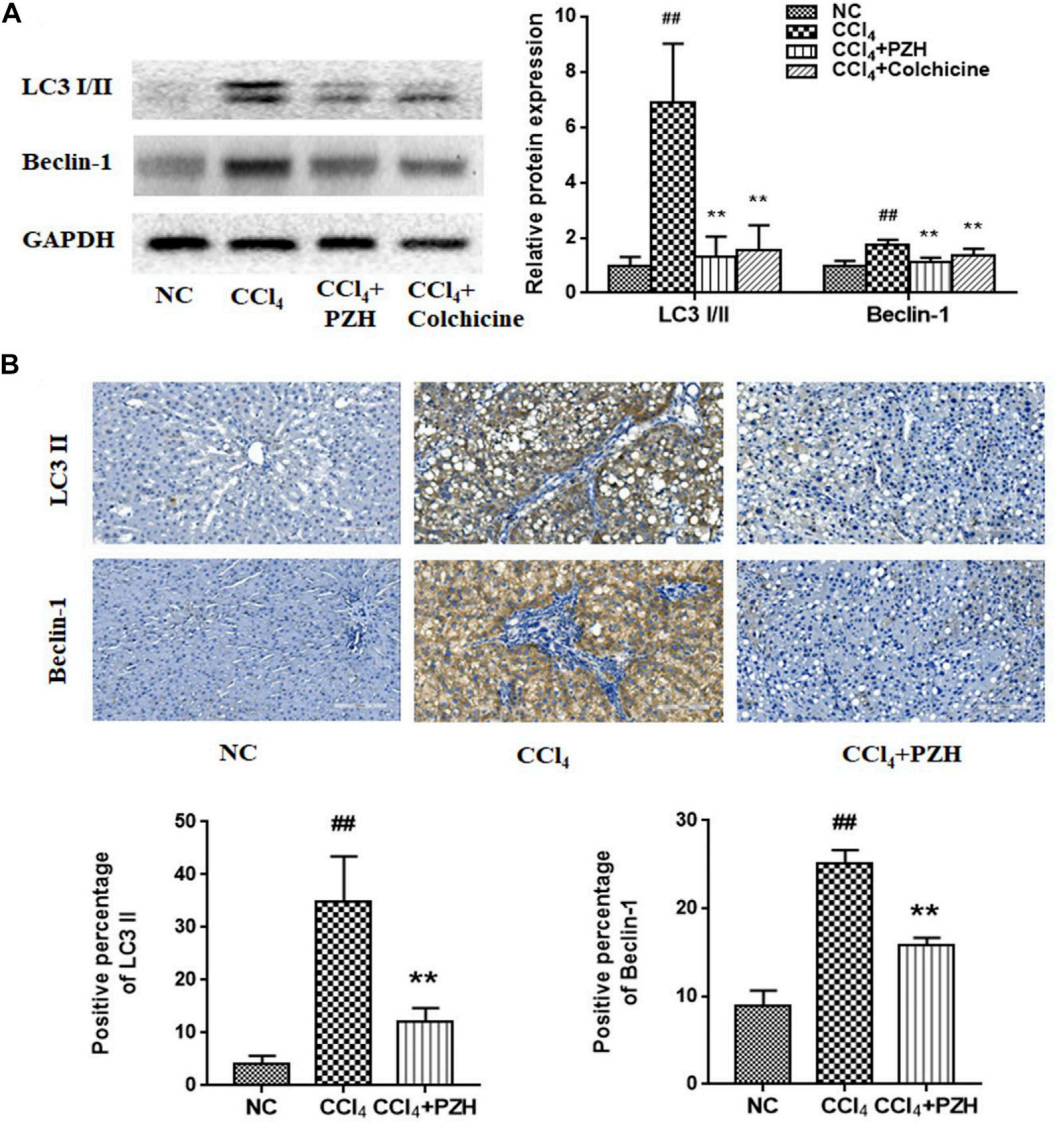
PZH suppressed CCl_4_-induced autophagy in liver fibrosis. **(A)** Western blot and densitometry analysis of LC3 and Beclin-1 protein levels; **(B)** immunohistochemical staining images and positive immunoreactivity analysis of LC3 II and Beclin 1 in liver sections (magnification×200). ^##^ *p* < 0.01 versus NC group; ^**^ *p* < 0.01 versus CCl_4_ group.

Furthermore, the alternation of liver autophagy was evaluated in each group. Western blot analysis displayed high expression of LC3 and Beclin-1in the liver fibrosis group against the normal group. Interestingly, expression levels were decreased in the other two treatment groups (Figure 2A). Moreover, IHC staining exhibited that the reactivity of LC3 and Beclin-1 was diffusely positive in the model group and was weakly positive in the PZH treatment group (Figure 2B) that was in line with the trend of proteins by means of western blot.

### Pien-Tze-Huang restrained transforming growth factor-β1-induced hepatic stellate cell activation and autophagy marker expression

**FIGURE 3:**
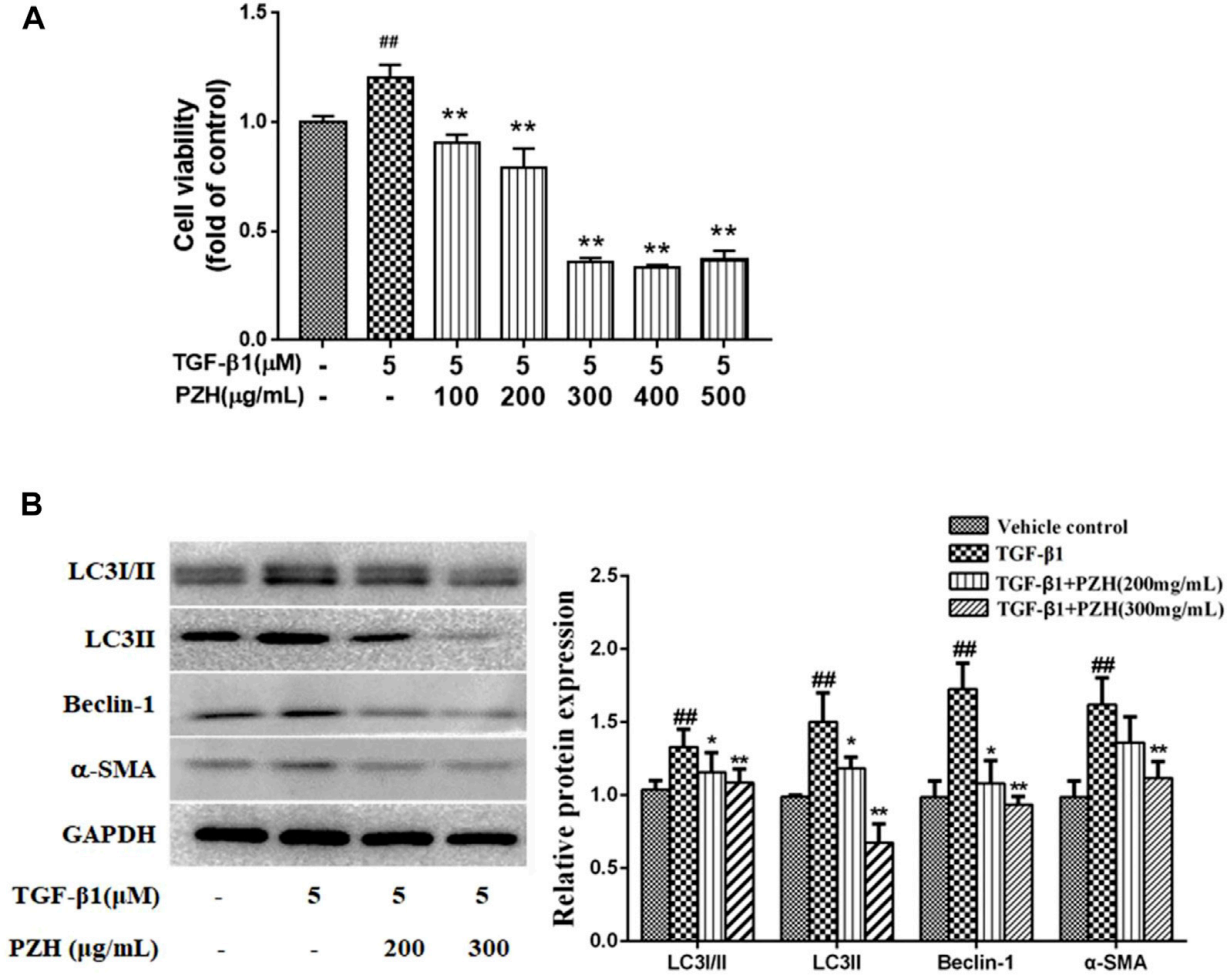
PZH restrained TGF-β1-induced HSC activation and autophagy marker expression *in vitro*. **(A)** MTT assay of TGF-β1-induced HSC viability under PZH pretreatment with serial concentrations of 100, 200, 300, 400, and 500 μg/ml; **(B)** Western blot and densitometry analysis of LC3, Beclin-1, and α-SMA protein levels in cultured HSC. GAPDH was used as an internal control. ^##^ *p* < 0.01 versus vehicle group; *p < 0.05, **p < 0.01 versus TGF-β1 group.

For further elucidating PZH’s impact on HSC autophagy, we employed recombinant TGF-β1 for inducing the activation of cultured HSC-T6 cells. Next, MTT assay revealed that HSC-T6 incubated with TGF-β1 for 48 h promoted cell proliferation that was suppressed after the PZH pretreatment. Also, we found that the inhibitory effect was dose-dependent linking between 100 and 300 μg/ml. It is noteworthy that the concentration of PZH is greater than 300 μg/ml, and its inhibitory effect not elevated the increase of dose (Figure 3A). Based on this, we selected 200 and 300 μg/ml as two dose groups of PZH for the subsequent Western blotting. As shown in Figure 3B, the expression of autophagy markers (LC3Ⅰ/Ⅱ, LC3Ⅱ, and Beclin-1) of HSC increased under TGF-β1 stimulation, accompanied by high expression of α-SMA. By contrast, preconditioning of PZH markedly curbed the expression of α-SMA, LC3Ⅰ/Ⅱ, LC3Ⅱ, and Beclin-1 in activated HSC in a dose-dependent manner.

### Pien-Tze-Huang mitigated carbon tetrachloride-induced liver fibrosis by transforming growth factor-β1/Smad2 signaling

**FIGURE 4:**
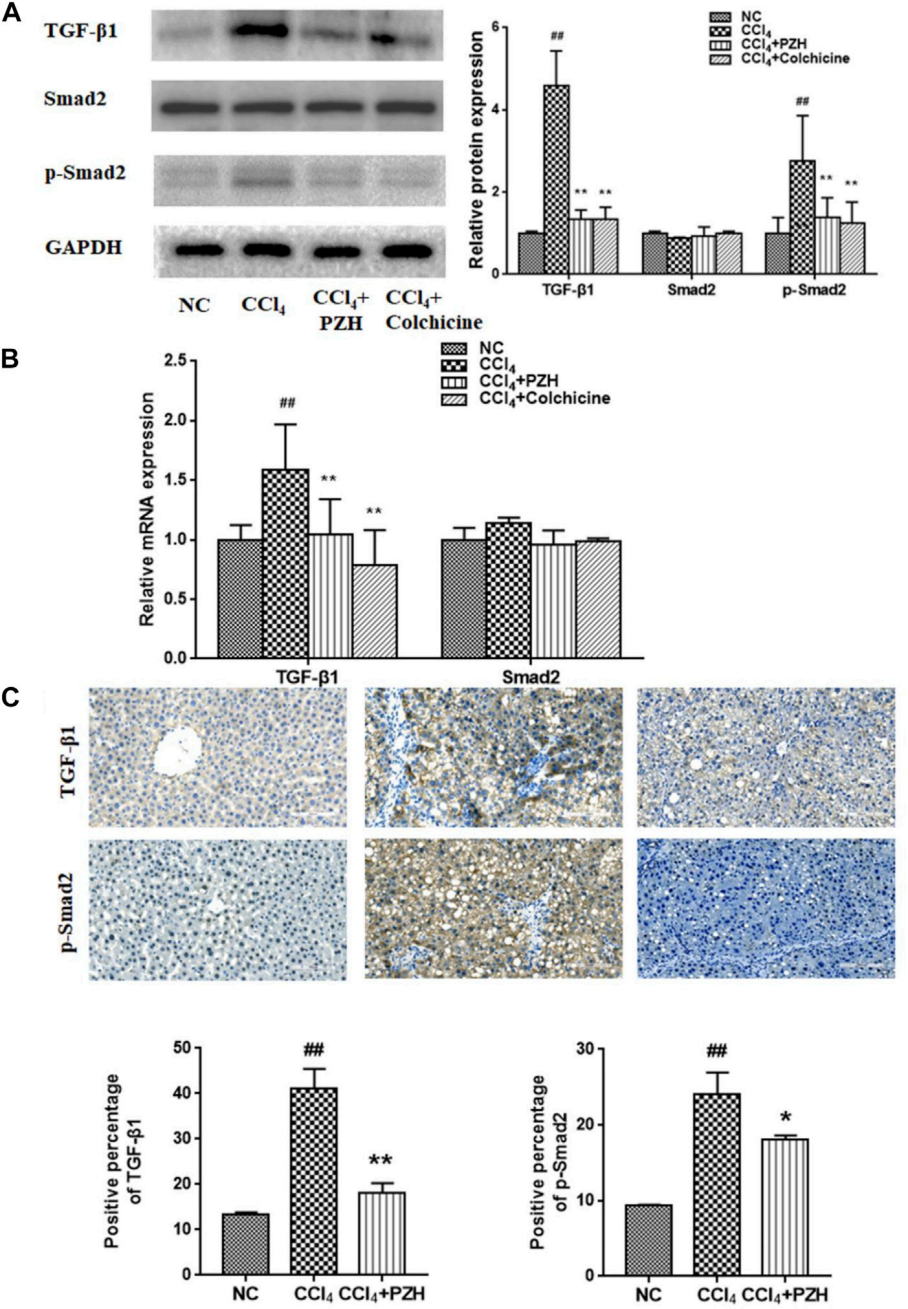
PZH mitigated CCl_4_-induced liver fibrosis *via* TGF-β1/Smad2 signaling. **(A)** Western blot and densitometry analysis of TGF-β1, Smad2, and p-Smad2 protein levels in liver tissues; **(B)** quantitative PCR detection of TGF-β1 and Smad2 mRNA levels; **(C)** immunohistochemical staining images and positive immunoreactivity analysis of TGF-β1and p-Smad2 in liver sections (magnification×200). ^##^ *p* < 0.01 versus NC group; *p < 0.05, **p < 0.01 versus CCl_4_ group.

TGF-β1 remains a powerful fibrogenic factor which can provoke the activation and proliferation of HSC upon liver lesions. The present study also investigated in detail at whether PZH had an impact on the expression of TGF-β1 and its downstream key transduction factor Smad2 during CCl_4_-induced liver fibrosis. Western blotting results showed an elevated expression of TGF-β1 and phosphorylated Smad2 (p-Smad2) in the fibrotic liver compared to that in a normal liver. In contrast, PZH or colchicine treatment was able to counteract the elevation of TGF-β1 and p-Smad2 expression under fibrogenesis (Figure 4A). Additionally, the expression of TGF-β1mRNA and proteins in each group showed a consistent trend, while the expression of Smad2 mRNA showed no significant difference (Figure 4B). In parallel, IHC results exhibited that the immunoreactivity of TGF-β1 and p-Smad2 was robust in the fibrosis model group and faint in the PZH treatment group (Figure 4C).

**FIGURE 5:**
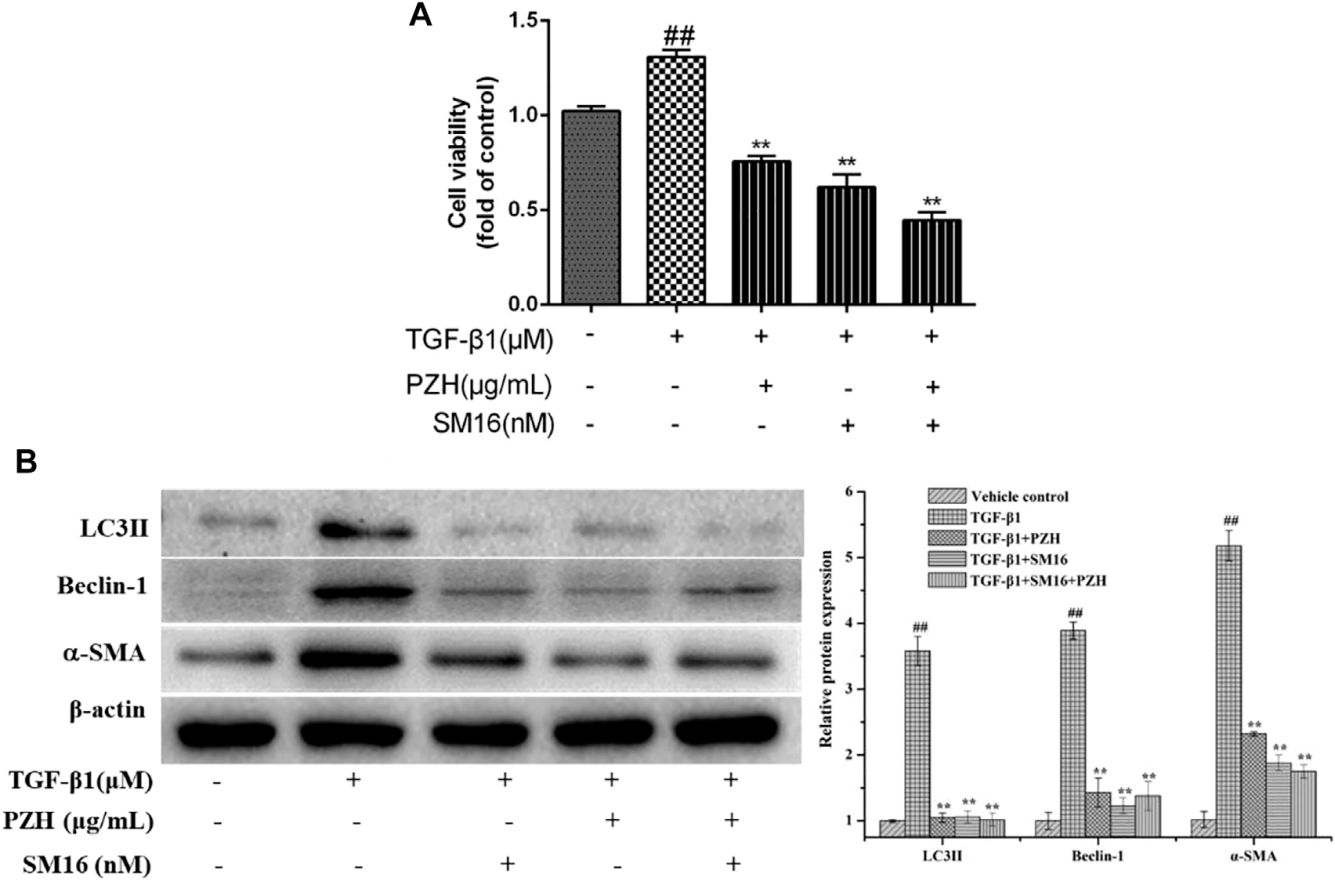
Effects of Pien-Tze-Huang on the proliferation and autophagy of HSC while the TGF-β1/Smad2 signaling pathway is blocked. **(A)** MTT assay of TGF-β1-induced HSC viability under SM16 (200 nM) and PZH (200 μg/ml) pretreatment; **(B)** Western blot and densitometry analysis of LC3, Beclin-1, and α-SMA protein levels in cultured HSC. β-actin was used as an internal control. ^##^ *p* < 0.01 versus vehicle group; *p < 0.05, **p < 0.01 versus TGF-β1 group.

Smad2 phosphorylation is a key mechanism in the mediation of autophagy activation. To further confirm the connection between Smad2 and PZH′s effect on autophagy, the activation of HSC and protein levels of autophagy markers (LC3Ⅱ and Beclin-1) was performed and the results showed that the protein levels of autophagy markers (LC3Ⅱ and Beclin-1) in TGF-β1-induced HSC cells were obviously increased, which was inhibited by SM16 that serves as a SMAD2 inhibitor (Figure 5B). In addition, the cell viability and α-SMA expression in the TGF-β1 group were higher than those in the control group and SM16 repressed these levels (Figures 5A,B). PZH shared the same effect as SM16.

## Discussion

Liver fibrosis is a sophisticated lesion implicated in multiple cells, pathways, and molecules (Sung et al., 2022). Interestingly, it remains liable to prevent or reverse fibrogenesis with “multi-targets” drugs (Gong et al., 2022). Accumulating evidence reported that TCM postures unique advantages of pharmacological action *via* multiple targets and holistic regulation (Li, 2020). In recent times, a variety of active compounds have been isolated and identified from PZH increasingly (Li et al., 2022b). Recently reported compounds are notoginsenoside (Cao et al., 2022), ginsenosides (Ramli et al., 2022), taurine (McGurk et al., 2022), citric acid and malic acid (Słupski et al., 2022), muscone (Fu et al., 2016) etc., which have been demonstrated to exhibit antioxidant, anti-inflammatory, and anti-tumor effects (Huang et al., 2019). It has been reported that PZH perhaps heals a wide range of liver injuries and inflammation (Fu et al., 2016; Qi et al., 2017; Li et al., 2018; Zhang et al., 2018). Owing to its history of around 500 years, PZH was listed as one of the Chinese National Protected TCM in the 1990s (Chen, 2021). Also, the major active components with their potential functions such as neuro-protecting effects, anti-cancer effects, and anti-inflammatory effects including most of the pharmacological effects had been validated and reported (Yan et al., 2017; Deng et al., 2020). We previously explored the therapeutic effect and potential effectiveness of PZH in countering liver fibrosis caused by chemical toxicity; however, its mechanism of action was not quite equivocal.

CCl_4_ is one of the most commonly employed chemical agents in establishing the liver fibrosis model in rodents (Yanguas et al., 2016). After exposure to CCl_4_, the progression of liver fibrosis is characterized by the activation of numerous HSCs, accompanied by hepatocellular necrosis and inflammation (Wu et al., 2022). The activated HSCs exhibit proliferation capacity and a myofibroblast-like phenotype which perhaps increase the expression of α-SMA, collagens, and a group of matrix enzymes (Li et al., 2022a). Mechanistically, CCl_4_ promotes the expression of SIRT3 and activity in the fibrotic liver and activate LX-2 cells (Li et al., 2022b). In the case of long-term injury and inflammation, the formation of liver fibrosis depends on two factors: in preference—the synthesis of fibrillar ECM components increases; on the other—the imbalance of MMPS/TIMPS reduces their degradation (Ginès et al., 2022). Earlier studies reported that MMP2 and MMP9 are primarily produced by activated HSC. Consequently, their release possibly increased in CCl_4_-induced liver fibrosis tissues, signifying that they primarily affect fibrogenesis rather than fibrolysis (Robert et al., 2016).

Next, TIMP1, an endogenous inhibitor of MMPs, remains to be secreted by activated HSC. Henceforth, TIMP1 binds and inhibits activated MMPs which in turn protect the newly synthesized collagen from being degraded by MMPs (Hemmann et al., 2007). In the present study, we revealed that PZH not only reduces liver damage caused by chemical toxicity but also improves liver function. Also, it slows down the development of liver fibrosis by blunting the activation of collagen-producing cells and regulating the equilibrium of matrix-degrading enzymes. Although the link between autophagy and hepatofibrosis is well-accepted, the role of autophagy is still complex as the liver harbors multiple parenchyma and mesenchymal cells. Allaire et al. (2019) reviewed the impact of autophagy on hepatofibrosis from a good perspective (Mallat et al., 2014). In short, the autophagy flux (i.e., the flux of material from the autophagosome into the lysosomal lumen) in the HSC is enhanced during liver fibrosis, and thus, accelerates the activation of the HSC and exacerbating fibrogenesis (Wu et al., 2022). In contrast, suppression of autophagy can curb HSC proliferation and facilitate HSC apoptosis.

Furthermore, contrary to the pro-fibrosis effect in the HSC, autophagy also protects hepatocytes by removing intracellular damage and abnormal aggregation of proteins (Yu et al., 2022). In addition, autophagy plays an anti-inflammatory role in reducing the release of inflammatory mediators in macro-autophagy (Liu et al., 2022). Generally, the functions of autophagy are multifaceted and cell-specific in the process of liver fibrosis (Jin et al., 2022). Therefore, we investigated whether PZH alleviated liver fibrosis by regulating autophagy. In the present study, we explored the autophagy level of the whole liver elevation after CCl_4_ induction. It was observed that the expression level of general autophagy was down-regulated after PZH treatment, and autophagosomes were detected occasionally. Furthermore, we focused on the changes of autophagy in activated HSC after PZH administration. As expected *in vitro*, PZH inhibited autophagy in TGF-β-induced HSC activation. According to previous studies, PZH will possibly induce the apoptosis of the HSC, so we speculated that the autophagy pathway might be a key pathway for PZH to induce the apoptosis of activated HSC. Growing evidence has shed light on the TGF-β1 that exerts multifunction pertaining cell growth, differentiation, apoptosis, and autophagy in a context-specific manner (Kiyono et al., 2009; Dewidar et al., 2019). The Smad pathway serves as canonical intracellular mediators of TGF-β1 signaling which then transfer into the nucleus to modulate numerous gene transcription processes. In terms of liver fibrosis, the TGF-β1/Smad pathway is involved in promoting the vitality of HSC in addition to the release of inflammatory mediators and the synthesis of the ECM.

The present findings showed that TGF-β1 induces the activation of cultured HSC and boosts the expression of autophagy-related genes. *In vivo*, TGF-β1/Smad2 signaling was up-regulated in the CCl_4_-induced hepatic fibrosis model, whereas PZH treatment curbed CCl_4_-induced high expression of TGF-β1 and p-Smad2. This result indicated that PZH might modulate autophagy through the TGF-β1/Smad signaling pathway. Recently, it has been reported that persistent phosphorylation of Smad2 is necessary for complete trans-differentiation of human fibroblasts, suggesting its relevance to HSC activation (Dewidar et al., 2019). Smad2 phosphorylation is also considered a key mechanism in the mediation of autophagy activation. As expected, PZH shared the same effect as SM16 inhibiting autophagy markers (LC3Ⅱ and Beclin-1) in this study. It is to be noted that in addition to the canonical TGF-β1/Smad pathway, Smad enables crosstalk with other signaling pathways, such as the NF-κB and MAPK signaling pathways (Luedde and Schwabe, 2011; Xu et al., 2016). Before this, we reported that PZH could attenuate liver fibrosis and prompt HSC apoptosis by inhibiting IκB-α, an inhibitor of NF-κB (Zheng et al., 2019). Taken together, our findings suggested that in the context of liver fibrosis, PZH could interact with multiple intracellular signaling pathways through TGF-β1 signaling and execute divergent functions, along with inhibiting HSC activation and diminishing fibrogenesis.

## Conclusion

To conclude, PZH exerted a beneficial role in combating toxicity-induced liver fibrosis and its molecular mechanisms tightly pertain to the accommodation of autophagy and TGF-β1/Smad pathways. Future studies are needed to clarify the key regulatory targets of these signaling pathways underlying PZH in the treatment of liver fibrosis.

## Data Availability

The raw data supporting the conclusion of this article will be made available by the authors, without undue reservation.

## Abbreviations

ATGs, autophagy-related genes; CCl_4_, carbon tetrachloride; ECM, extracellular matrix; H&E, hematoxylin-eosin; HSC, hepatic stellate cell; IHC, immunohistochemistry; LC3, light chain 3; MMP, matrix metalloproteinase 2; NF-κB, nuclear factor kappa-light-chain-enhancer of activated B; PZH, Pien-Tze-Huang; qPCR, quantitative polymerase chain reaction; TCM, traditional Chinese medicine; TGFR, TGF receptor; TGF-β1, transforming growth factor-β1; TIMP, tissue inhibitor of matrix metalloproteinase; TNF, tumor necrosis factor; VG, Van Gieson; α-SMA, α-smooth muscle actin.
