# Supplementary figures and images for: Pien-Tze-Huang alleviates CCl4-induced liver fibrosis through the inhibition of HSC autophagy and the TGF-β1/Smad2 pathway
Source: Front Pharmacol. 2022 Sep 16;13:937484. doi: 10.3389/fphar.2022.937484 (PMC9523731; doi:10.3389/fphar.2022.937484)

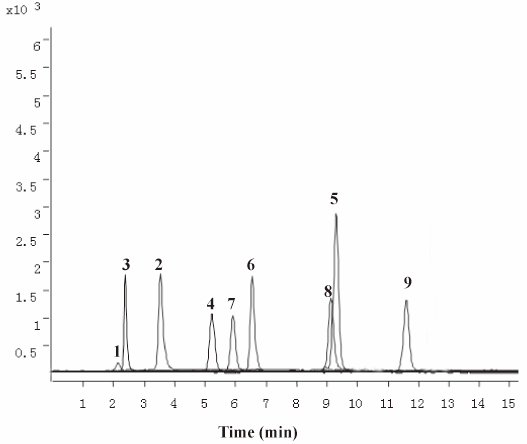

Supplement: Supplementary file 1 [file Image1.tiff]

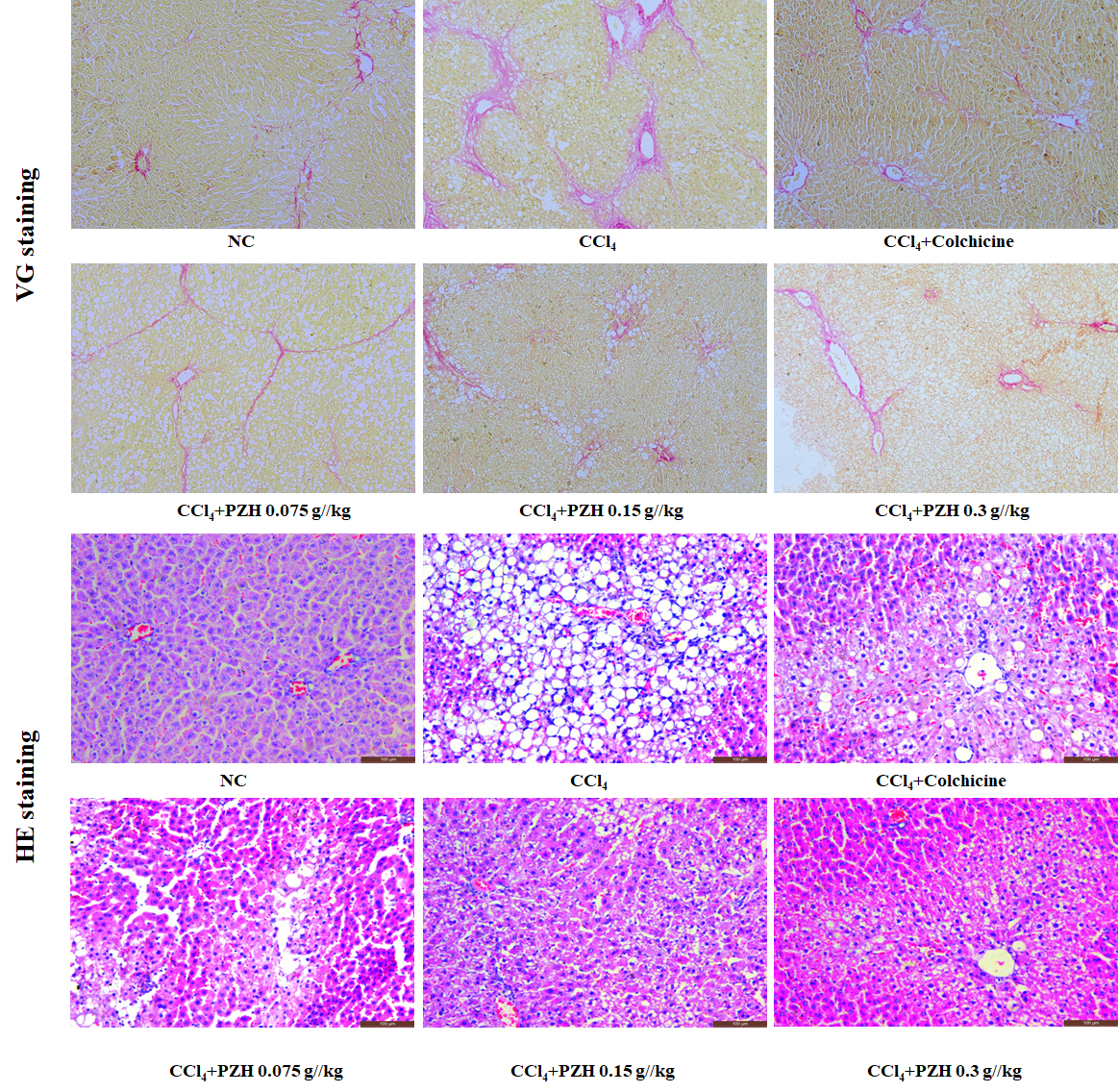

Supplement: Supplementary file 2 [file Image2.tif]
